# *catena*-Poly[[manganese(III)-bis­{μ-2-[(2-hy­droxy­eth­yl)imino­meth­yl]-6-meth­oxy­phenolato-κ^3^ *O* ^1^,*N*:*O* ^2^;κ^3^ *O* ^2^:*N*,*O* ^1^}] iodide]

**DOI:** 10.1107/S1600536813012695

**Published:** 2013-05-18

**Authors:** Svitlana R. Petrusenko, Oleg M. Stetsyuk, Irina V. Omelchenko

**Affiliations:** a Department of Inorganic Chemistry,Taras Shevchenko National University of Kyiv, 64/13 Volodymyrs’ka St, Kyiv 01601, Ukraine; b SSI "Institute for Single Crystals" National Academy of Sciences of Ukraine, 60 Lenin Ave., Kharkiv 61001, Ukraine

## Abstract

In the title one-dimensional coordination polymer, {[Mn(C_10_H_12_NO_3_)_2_]I}_*n*_, the potentially tetra­dentate (*O*,*O*,*O*,*N*) 2-[(2-hy­droxy­eth­yl)imino­meth­yl]-6-meth­oxy­phenol (H_2_ *L*) ligands are mono-deprotonated (as H*L* ^−^) and coordinated by the metal ions in a tridentate chelate-bridging fashion [2.01_1_1_1_1_2_]. The Mn^III^ atom possesses a distorted *trans*-MnO_4_N_2_ octa­hedral coordination environment. The bridging ligands lead to [010]-chain polymeric cations {[Mn(H*L*)_2_]^+^}_*n*_ in the crystal. The charge-balancing iodide ions are disordered over two sites in a 0.690 (2):0.310 (2) ratio and a weak O—H⋯I hydrogen bond occurs. The crystal studied was found to be a racemic twin.

## Related literature

For the related structure of {[Mn(C_9_H_10_NO_2_)_2_]Cl}_*n*_, see: Zhang *et al.* (2005 ▶). For further synthetic details, see: Babich *et al.* (1996 ▶); Vinogradova *et al.* (2002 ▶); Makhankova *et al.* (2002 ▶); Nesterov *et al.* (2012 ▶); Chygorin *et al.* (2012 ▶). For bond-valence sum calculations, see: Brown & Altermatt (1985 ▶). For coordination mode notation, see: Coxall *et al.* (2000 ▶).
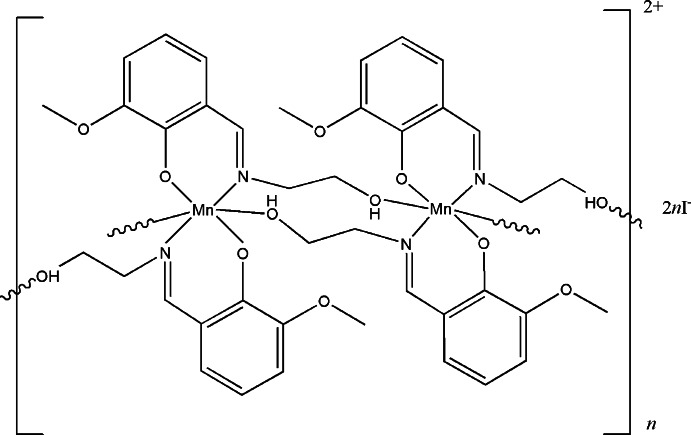


## Experimental

### 

#### Crystal data

[Mn(C_10_H_12_NO_3_)_2_]I
*M* *_r_* = 570.25
Orthorhombic, 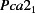

*a* = 18.880 (2) Å
*b* = 5.8979 (10) Å
*c* = 20.916 (2) Å
*V* = 2329.1 (5) Å^3^
*Z* = 4
Mo *K*α radiation
μ = 1.93 mm^−1^
*T* = 298 K
0.40 × 0.20 × 0.20 mm

#### Data collection

Agilent Xcalibur Sapphire3 diffractometer
Absorption correction: multi-scan (*CrysAlis PRO*; Agilent, 2011 ▶) *T*_min_ = 0.513, *T*_max_ = 0.699
7841 measured reflections
4752 independent reflections
2094 reflections with *I* > 2σ(*I*)
*R*_int_ = 0.078

#### Refinement

*R*[*F* ^2^ > 2σ(*F* ^2^)] = 0.076
*wR*(*F* ^2^) = 0.173
*S* = 0.92
4752 reflections
282 parameters
1 restraint
H-atom parameters constrained
Δρ_max_ = 1.49 e Å^−3^
Δρ_min_ = −0.66 e Å^−3^
Absolute structure: Flack (1983 ▶), 1276 Friedel pairs
Flack parameter: 0.59 (3)

### 

Data collection: *CrysAlis CCD* (Agilent, 2011 ▶); cell refinement: *CrysAlis RED* (Agilent, 2011 ▶); data reduction: *CrysAlis RED*; program(s) used to solve structure: *SHELXS97* (Sheldrick, 2008 ▶); program(s) used to refine structure: *OLEX2* (Dolomanov *et al.*, 2009 ▶); molecular graphics: *SHELXTL* (Sheldrick, 2008 ▶); software used to prepare material for publication: *publCIF* (Westrip, 2010 ▶).

## Supplementary Material

Crystal structure: contains datablock(s) I, global. DOI: 10.1107/S1600536813012695/hb7060sup1.cif

Structure factors: contains datablock(s) I. DOI: 10.1107/S1600536813012695/hb7060Isup2.hkl

Additional supplementary materials:  crystallographic information; 3D view; checkCIF report

## Figures and Tables

**Table 1:** Selected bond lengths (Å)

Mn1—O3	1.829 (8)
Mn1—O1	1.849 (8)
Mn1—N1	2.035 (10)
Mn1—N2	2.061 (9)
Mn1—O6^i^	2.247 (8)
Mn1—O5^ii^	2.315 (8)

Symmetry codes: (i) 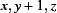; (ii) 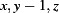.

**Table 2:** Hydrogen-bond geometry (Å, °)

*D*—H⋯*A*	*D*—H	H⋯*A*	*D*⋯*A*	*D*—H⋯*A*
O5—H5⋯I1*A* ^iii^	0.86	2.86	3.488 (8)	131

Symmetry code: (iii) 
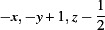
.

**Fig. 1.**
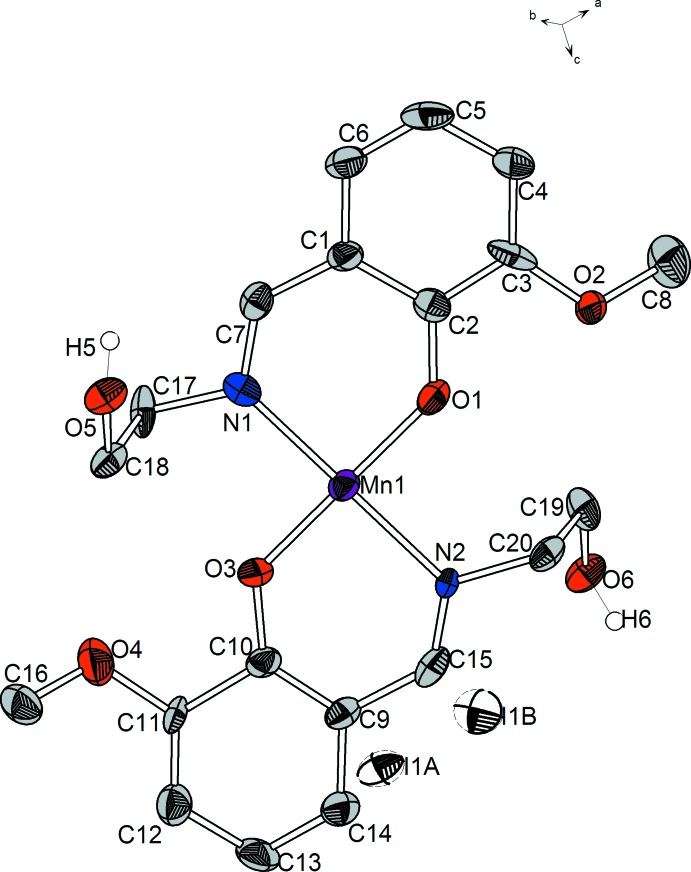
The asymmetric unit of (I) with 30% probability ellipsoids for non-H atoms. C—H hydrogen atoms are ommited for clarity. Iodide ion is disodered in two positions I1A and I1B with occupancy ratio 0.69:0.31, respectively.

**Fig. 2.**
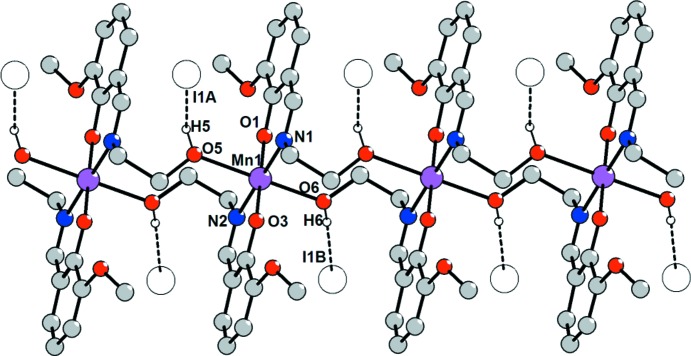
Fragment of chain polymeric structure of (I).

**Fig. 3.**
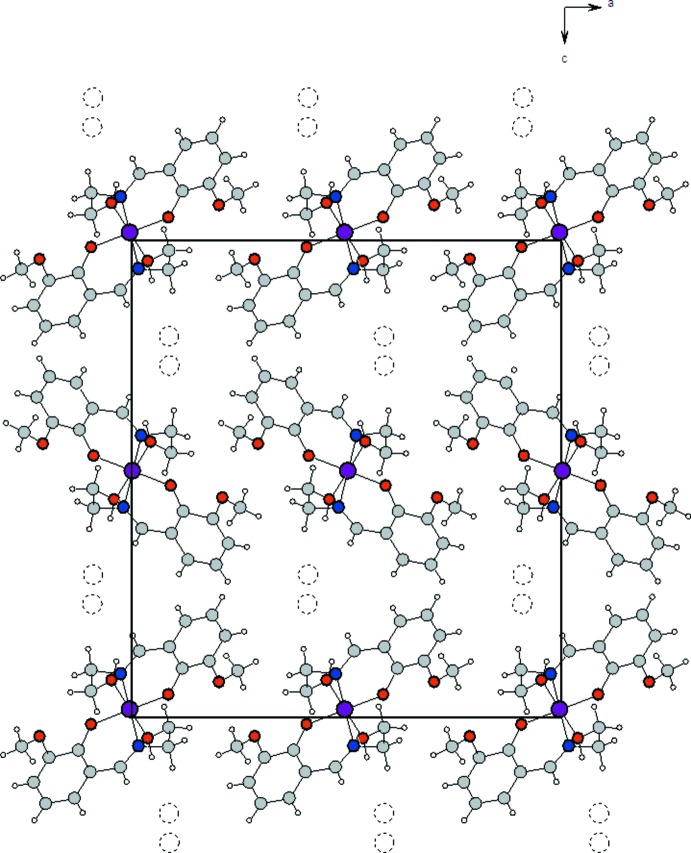
Packing of (I) viewed down the (010) direction.

## supplementary crystallographic information

### Comment

Developing the "direct synthesis" approach (Babich *et al.*, 1996; Vinogradova *et al.*, 2002; Makhankova *et al.*, 2002), our research group are now interested in the preparation of manganese-based heterometallic "salen-type" Schiff bases complexes as promising objects for search and production of new materials with useful properties. Synthesis from metal powders as reagents has been recently demonstrated to be an alternative and efficient way to similar Fe/Co complexes (Nesterov *et al.*, 2012; Chygorin *et al.*, 2012). But in some cases instead of heterometallic compounds we can obtain monometallic ones or a mixture of different compounds as more thermodinamically favorable products in selected conditions. Such a case is observed with the investigated system

Mn^0^ – Fe^0^ – {3(*o*-vanillin) – 3(Hea)} – 2NH_4_I – CH_3_OH (t=50 ^0^C, in open air),

where *o*-vanillin = 2-hydroxy-3-methoxybenzaldehyde; Hea = 2-aminoethanol;

from which the new polymeric complex [Mn(HL)_2_]I (H_2_L = 2-hydroxyiminomethyl-6-methoxyphenol), (I), was isolated.

The total dissolution of metal powders was observed within 6 h resulting into intensive dark brown solution. The block brown crystalls precipitate after 24 h with 45% yield. Interestingly that the stechiometric system

Mn^0^ – {2(*o*-vanillin) – 2(Hea)} – NH_4_I – CH_3_OH

produces the same complex, but the dissolution of metal powders is longer (more than 7 h) and the yield is lower (26%).

The {[Mn(HL)_2_]}_n_ unit (Fig.1) demonstrates the [O_4_N_2_] coordination environment with a distorted octahedral geometry around the central atom. The metal assignment and its oxidation state were confirmed by considering coordination bond lengths, existence of Jahn-Teller elongation and bond valence sum calculation [BVS(Mn) = 3.07 (Brown & Altermatt, 1985)].

The one-dimensional polymeric structure of the crystal (Fig. 2) is realised by means of chelate-bridging function of the ligand, [2.01_1_1_1_1_2_] by Harris notation (Coxall *et al.*, 2000), which is firstly observed for H_2_L.

The disodered iodide anions occupy channels between polymeric cationic chains joining to them through weak O–H···I hydrogen bonds (H···I 2.62 - 2.86 Å, O—H···I 131 - 172°) forming neutral chains along (010) direction (Fig. 2, 3). It is worth noting that in the similar complex {[Mn(HL')_2_]Cl}_n_, where H_2_L'= 2-[(2-hydroxyethyl)iminomethyl]-phenol (Zhang *et al.*, 2005), the polymeric chains interlink with numerous O–H···Cl hydrogen bonds and C–H···π contacts yielding two-dimensional network. This difference can be caused by at least two factors: (1) greater electronegativeness and notably smaller radius of Cl^-^ anions, and (2) absence of methoxy-group, and so additional steric hindrance, in H_2_L'.

### Experimental

2-Aminoethanol (0.18 g, 3 mmol) and *o*-vanillin (0.457 g, 3 mmol) were added to 30 ml of methanol and stirred magnetically for 15 min until the colour of the solution turned in yellow. After manganese powder (0.055 g, 1 mmol), iron powder (0.058 g, 1 mmol) and NH_4_I (0.29 g, 2 mmol) were added to the solution, the reaction mixture was stirred at 50°C for *ca* 6 h. Almost total dissolution of metal powders was observed. Dark brown crystals that precipitated after 1 day were collected by filtration and dried in air; yield 45%. Elemental analysis for C_20_H_24_I_2_MnN_2_O_6_ (Mr = 570.26). Calcd: Mn, 9.63%. Found: Mn, 9.3%. IR(KBr, cm^-1^): 3457(*b*), 3411(w), 3248(*b*), 3193(w), 2993(w), 2939(w), 2830(w), 1615(*s*), 1552(*m*), 1459(*s*), 1443(*s*), 1406(*m*), 1343(*m*), 1316(*s*), 1243(*s*), 1216(*m*) 1161(w), 1107(w), 1071(*m*), 1017(*m*), 980(*m*), 898(w), 871(*m*), 789(w), 726(*s*), 635(*m*), 599(w), 526(w), 454(w), 417(w).

### Refinement

All H atoms were placed in idealized positions (C–H = 0.93 – 0.97 Å, O–H = 0.86 Å) and constrained to ride on their parent atoms, with *U*_iso_ = 1.2Ueq (except *U*_iso_ = 1.5Ueq for methyl and hydroxyl groups). It should be noted that the mean value of the |E^2^-1| is 0.816 and the structure can be solved in both polar (*Pca2_1_*) and centrosymmetric (*Pbca*) groups, however, the *R_1_* value in the latter case is consistently higher (*ca*. 0.24) that in the former (less than 0.10), so the noncentrosymmetric space group is believed to be the correct choice. Flack parameter value (Flack, 1983) of 0.48 (5) was obtained in the final structure factor calculation, indicating a racemic twin. for Futher full-matrix refinement of the Flack parameter slightly improved the agreement index *R* (from 0.077 to 0.076). Content of the the major component in the refined racemic twin structure is 59 (3)%. Iodine atom was found to be disordered over two sites with with occupancy factors 0.69 and 0.31.

### Crystal data

**Table:**

[Mn(C_10_H_12_NO_3_)_2_]I	*F*(000) = 1136
*M**_r_* = 570.25	*D*_x_ = 1.626 Mg m^−^^3^
Orthorhombic, *P**c**a*2_1_	Mo *K*α radiation, λ = 0.71073 Å
Hall symbol: P 2c -2ac	Cell parameters from 861 reflections
*a* = 18.880 (2) Å	θ = 2.9–32.3°
*b* = 5.8979 (10) Å	µ = 1.93 mm^−^^1^
*c* = 20.916 (2) Å	*T* = 298 K
*V* = 2329.1 (5) Å^3^	Block, brown
*Z* = 4	0.40 × 0.20 × 0.20 mm

### Data collection

**Table:**

Agilent Xcalibur Sapphire3 diffractometer	4752 independent reflections
Radiation source: Enhance (Mo) X-ray Source	2094 reflections with *I* > 2σ(*I*)
Graphite monochromator	*R*_int_ = 0.078
Detector resolution: 16.1827 pixels mm^-1^	θ_max_ = 30.0°, θ_min_ = 3.5°
ω scans	*h* = −26→10
Absorption correction: multi-scan (*CrysAlis PRO*; Agilent, 2011)	*k* = −6→8
*T*_min_ = 0.513, *T*_max_ = 0.699	*l* = −29→28
7841 measured reflections	

### Refinement

**Table:**

Refinement on *F*^2^	Secondary atom site location: difference Fourier map
Least-squares matrix: full	Hydrogen site location: inferred from neighbouring sites
*R*[*F*^2^ > 2σ(*F*^2^)] = 0.076	H-atom parameters constrained
*wR*(*F*^2^) = 0.173	*w* = 1/[σ^2^(*F*_o_^2^) + (0.055*P*)^2^] where *P* = (*F*_o_^2^ + 2*F*_c_^2^)/3
*S* = 0.92	(Δ/σ)_max_ < 0.001
4752 reflections	Δρ_max_ = 1.49 e Å^−^^3^
282 parameters	Δρ_min_ = −0.66 e Å^−^^3^
1 restraint	Absolute structure: Flack (1983), 1276 Friedel pairs
Primary atom site location: structure-invariant direct methods	Flack parameter: 0.59 (3)

### Special details

**Table:**

Experimental. Absorption correction: CrysAlis PRO (Agilent, 2011). Empirical absorption correction using spherical harmonics, implemented in SCALE3 ABSPACK scaling algorithm.
Geometry. All e.s.d.'s (except the e.s.d. in the dihedral angle between two l.s. planes) are estimated using the full covariance matrix. The cell e.s.d.'s are taken into account individually in the estimation of e.s.d.'s in distances, angles and torsion angles; correlations between e.s.d.'s in cell parameters are only used when they are defined by crystal symmetry. An approximate (isotropic) treatment of cell e.s.d.'s is used for estimating e.s.d.'s involving l.s. planes.
Refinement. Refinement of *F*^2^ against ALL reflections. The weighted *R*-factor *wR* and goodness of fit *S* are based on *F*^2^, conventional *R*-factors *R* are based on *F*, with *F* set to zero for negative *F*^2^. The threshold expression of *F*^2^ > σ(*F*^2^) is used only for calculating *R*-factors(gt) *etc*. and is not relevant to the choice of reflections for refinement. *R*-factors based on *F*^2^ are statistically about twice as large as those based on *F*, and *R*- factors based on ALL data will be even larger.

### Fractional atomic coordinates and isotropic or equivalent isotropic displacement parameters (Å^2^)

**Table:**

	*x*	*y*	*z*	*U*_iso_*/*U*_eq_	Occ. (<1)
I1A	0.08961 (6)	0.43638 (19)	0.26350 (5)	0.0583 (4)	0.690 (2)
I1B	0.08839 (17)	−0.0686 (6)	0.20195 (17)	0.0752 (12)	0.310 (2)
Mn1	−0.00346 (8)	−0.2483 (4)	−0.01586 (13)	0.0360 (3)	
O1	0.0867 (4)	−0.3014 (13)	−0.0471 (4)	0.046 (2)	
N1	−0.0240 (4)	−0.0371 (15)	−0.0905 (5)	0.036 (2)	
C1	0.0895 (7)	−0.0934 (19)	−0.1467 (5)	0.045 (3)	
N2	0.0178 (4)	−0.4584 (16)	0.0605 (4)	0.036 (2)	
O2	0.2049 (4)	−0.5108 (13)	−0.0732 (4)	0.057 (2)	
C2	0.1194 (6)	−0.2539 (19)	−0.1020 (6)	0.041 (3)	
O3	−0.0923 (4)	−0.1935 (15)	0.0153 (4)	0.049 (2)	
C3	0.1822 (6)	−0.354 (3)	−0.1174 (7)	0.058 (4)	
O4	−0.2124 (5)	0.0167 (19)	0.0405 (6)	0.095 (4)	
C4	0.2190 (7)	−0.297 (2)	−0.1702 (6)	0.060 (4)	
H4	0.2633	−0.3603	−0.1782	0.072*	
O5	−0.0455 (4)	0.4550 (13)	−0.0775 (4)	0.048 (2)	
H5	−0.0337	0.4208	−0.1158	0.072*	
C5	0.1889 (7)	−0.138 (2)	−0.2134 (6)	0.067 (4)	
H5A	0.2124	−0.1046	−0.2514	0.081*	
O6	0.0391 (4)	−0.9640 (12)	0.0445 (4)	0.047 (2)	
H6	0.0484	−0.9805	0.0846	0.071*	
C6	0.1268 (7)	−0.035 (2)	−0.2004 (6)	0.053 (3)	
H6A	0.1093	0.0758	−0.2279	0.063*	
C7	0.0196 (6)	0.000 (2)	−0.1382 (6)	0.042 (3)	
H7	0.0037	0.0981	−0.1700	0.050*	
C8	0.2479 (9)	−0.695 (2)	−0.0966 (9)	0.078 (5)	
H8A	0.2609	−0.7920	−0.0616	0.117*	
H8B	0.2900	−0.6354	−0.1162	0.117*	
H8C	0.2216	−0.7807	−0.1275	0.117*	
C9	−0.0945 (6)	−0.3948 (19)	0.1152 (5)	0.040 (3)	
C10	−0.1217 (6)	−0.256 (2)	0.0695 (6)	0.039 (3)	
C11	−0.1901 (6)	−0.1460 (19)	0.0850 (6)	0.041 (3)	
C12	−0.2257 (6)	−0.200 (2)	0.1367 (7)	0.056 (4)	
H12	−0.2695	−0.1327	0.1443	0.067*	
C13	−0.1983 (6)	−0.358 (2)	0.1810 (8)	0.065 (4)	
H13	−0.2246	−0.4019	0.2165	0.078*	
C14	−0.1321 (7)	−0.445 (2)	0.1705 (6)	0.060 (4)	
H14	−0.1119	−0.5401	0.2010	0.072*	
C15	−0.0250 (6)	−0.496 (2)	0.1054 (6)	0.048 (3)	
H15	−0.0105	−0.6015	0.1359	0.058*	
C16	−0.2537 (8)	0.190 (2)	0.0618 (7)	0.065 (4)	
H16A	−0.2654	0.2880	0.0267	0.098*	
H16B	−0.2285	0.2746	0.0937	0.098*	
H16C	−0.2964	0.1298	0.0800	0.098*	
C17	−0.0928 (5)	0.0769 (15)	−0.0985 (7)	0.049 (3)	
H17A	−0.1310	−0.0249	−0.0869	0.059*	
H17B	−0.0993	0.1225	−0.1427	0.059*	
C18	−0.0929 (6)	0.2899 (19)	−0.0539 (6)	0.044 (3)	
H18A	−0.1403	0.3529	−0.0517	0.053*	
H18B	−0.0789	0.2458	−0.0110	0.053*	
C19	0.0885 (6)	−0.7926 (19)	0.0222 (7)	0.048 (4)	
H19A	0.0769	−0.7521	−0.0215	0.058*	
H19B	0.1360	−0.8551	0.0224	0.058*	
C20	0.0869 (5)	−0.5841 (19)	0.0628 (5)	0.040 (3)	
H20A	0.1244	−0.4829	0.0491	0.048*	
H20B	0.0966	−0.6267	0.1068	0.048*	

### Atomic displacement parameters (Å^2^)

**Table:**

	*U*^11^	*U*^22^	*U*^33^	*U*^12^	*U*^13^	*U*^23^
I1A	0.0828 (7)	0.0563 (6)	0.0359 (5)	−0.0091 (7)	−0.0043 (9)	−0.0054 (10)
I1B	0.087 (2)	0.0596 (19)	0.079 (2)	−0.0057 (18)	0.011 (2)	−0.002 (2)
Mn1	0.0441 (6)	0.0298 (5)	0.0340 (6)	0.0027 (6)	0.0010 (7)	0.0055 (6)
O1	0.054 (5)	0.049 (5)	0.036 (4)	0.008 (4)	−0.007 (5)	0.010 (4)
N1	0.039 (5)	0.027 (5)	0.042 (5)	−0.009 (4)	0.005 (5)	−0.015 (5)
C1	0.070 (8)	0.034 (6)	0.031 (5)	0.014 (6)	0.010 (7)	0.000 (5)
N2	0.038 (5)	0.038 (5)	0.033 (5)	0.017 (4)	0.003 (4)	0.014 (5)
O2	0.079 (5)	0.051 (5)	0.040 (5)	0.039 (4)	0.013 (5)	0.008 (5)
C2	0.044 (6)	0.038 (7)	0.043 (7)	−0.003 (5)	0.005 (6)	−0.007 (6)
O3	0.056 (5)	0.054 (6)	0.038 (5)	0.011 (4)	0.014 (5)	0.015 (5)
C3	0.044 (7)	0.087 (10)	0.044 (7)	−0.012 (7)	0.023 (6)	−0.027 (8)
O4	0.075 (6)	0.146 (11)	0.065 (7)	0.057 (7)	−0.009 (6)	−0.006 (8)
C4	0.069 (8)	0.077 (11)	0.034 (7)	0.018 (8)	0.011 (7)	−0.013 (8)
O5	0.069 (5)	0.043 (5)	0.033 (4)	−0.011 (4)	−0.010 (4)	−0.008 (5)
C5	0.097 (10)	0.076 (10)	0.028 (6)	−0.005 (9)	0.020 (7)	−0.011 (7)
O6	0.069 (5)	0.030 (4)	0.043 (4)	−0.010 (4)	−0.009 (5)	0.006 (5)
C6	0.076 (8)	0.048 (7)	0.035 (6)	0.004 (7)	0.008 (7)	0.003 (7)
C7	0.054 (7)	0.028 (7)	0.044 (7)	0.002 (5)	−0.008 (6)	−0.001 (6)
C8	0.085 (8)	0.043 (8)	0.106 (15)	0.012 (7)	0.006 (11)	−0.004 (10)
C9	0.045 (6)	0.041 (7)	0.035 (5)	−0.008 (5)	−0.003 (6)	0.010 (6)
C10	0.051 (7)	0.042 (7)	0.025 (5)	0.002 (5)	−0.003 (6)	−0.004 (6)
C11	0.040 (6)	0.041 (7)	0.041 (6)	0.011 (5)	−0.014 (6)	0.013 (6)
C12	0.048 (7)	0.061 (10)	0.057 (9)	0.006 (7)	−0.009 (7)	0.002 (9)
C13	0.058 (7)	0.065 (9)	0.071 (10)	0.002 (7)	0.036 (8)	0.018 (8)
C14	0.078 (8)	0.062 (9)	0.040 (7)	0.020 (7)	0.007 (7)	0.004 (8)
C15	0.063 (8)	0.042 (8)	0.039 (6)	0.000 (6)	−0.022 (7)	0.007 (6)
C16	0.067 (8)	0.069 (10)	0.059 (9)	0.016 (7)	0.010 (8)	−0.016 (9)
C17	0.029 (5)	0.016 (5)	0.102 (10)	−0.005 (4)	−0.018 (7)	0.016 (7)
C18	0.046 (6)	0.049 (8)	0.037 (6)	−0.010 (6)	−0.010 (6)	0.011 (6)
C19	0.048 (7)	0.025 (7)	0.072 (10)	0.008 (5)	0.006 (7)	−0.010 (7)
C20	0.042 (5)	0.048 (7)	0.030 (5)	−0.010 (5)	−0.018 (6)	0.005 (6)

### Geometric parameters (Å, º)

**Table:**

Mn1—O3	1.829 (8)	O6—H6	0.8625
Mn1—O1	1.849 (8)	C6—H6A	0.9300
Mn1—N1	2.035 (10)	C7—H7	0.9300
Mn1—N2	2.061 (9)	C8—H8A	0.9600
Mn1—O6^i^	2.247 (8)	C8—H8B	0.9600
Mn1—O5^ii^	2.315 (8)	C8—H8C	0.9600
O1—C2	1.335 (14)	C9—C10	1.358 (15)
N1—C7	1.312 (14)	C9—C14	1.391 (16)
N1—C17	1.473 (13)	C9—C15	1.457 (17)
C1—C6	1.371 (16)	C10—C11	1.482 (16)
C1—C7	1.443 (16)	C11—C12	1.312 (17)
C1—C2	1.445 (16)	C12—C13	1.414 (19)
N2—C15	1.259 (15)	C12—H12	0.9300
N2—C20	1.502 (13)	C13—C14	1.369 (16)
O2—C3	1.376 (17)	C13—H13	0.9300
O2—C8	1.442 (14)	C14—H14	0.9300
C2—C3	1.363 (16)	C15—H15	0.9300
O3—C10	1.315 (13)	C16—H16A	0.9600
C3—C4	1.349 (18)	C16—H16B	0.9600
O4—C16	1.361 (14)	C16—H16C	0.9600
O4—C11	1.401 (14)	C17—C18	1.566 (16)
C4—C5	1.421 (18)	C17—H17A	0.9700
C4—H4	0.9300	C17—H17B	0.9700
O5—C18	1.412 (12)	C18—H18A	0.9700
O5—Mn1^i^	2.315 (8)	C18—H18B	0.9700
O5—H5	0.8547	C19—C20	1.496 (17)
C5—C6	1.348 (17)	C19—H19A	0.9700
C5—H5A	0.9300	C19—H19B	0.9700
O6—C19	1.453 (13)	C20—H20A	0.9700
O6—Mn1^ii^	2.247 (8)	C20—H20B	0.9700
			
O3—Mn1—O1	179.5 (5)	O2—C8—H8C	109.5
O3—Mn1—N1	89.5 (4)	H8A—C8—H8C	109.5
O1—Mn1—N1	90.5 (3)	H8B—C8—H8C	109.5
O3—Mn1—N2	90.5 (4)	C10—C9—C14	121.4 (11)
O1—Mn1—N2	89.6 (3)	C10—C9—C15	119.3 (10)
N1—Mn1—N2	179.2 (5)	C14—C9—C15	119.2 (11)
O3—Mn1—O6^i^	89.8 (4)	O3—C10—C9	128.1 (11)
O1—Mn1—O6^i^	89.8 (3)	O3—C10—C11	115.6 (10)
N1—Mn1—O6^i^	92.4 (3)	C9—C10—C11	116.1 (11)
N2—Mn1—O6^i^	86.8 (3)	C12—C11—O4	124.0 (10)
O3—Mn1—O5^ii^	91.0 (4)	C12—C11—C10	121.4 (11)
O1—Mn1—O5^ii^	89.5 (3)	O4—C11—C10	114.6 (10)
N1—Mn1—O5^ii^	88.3 (3)	C11—C12—C13	120.8 (11)
N2—Mn1—O5^ii^	92.5 (4)	C11—C12—H12	119.6
O6^i^—Mn1—O5^ii^	179.0 (4)	C13—C12—H12	119.6
C2—O1—Mn1	134.0 (7)	C14—C13—C12	118.6 (12)
C7—N1—C17	113.0 (10)	C14—C13—H13	120.7
C7—N1—Mn1	124.6 (7)	C12—C13—H13	120.7
C17—N1—Mn1	122.3 (8)	C13—C14—C9	121.2 (13)
C6—C1—C7	118.3 (12)	C13—C14—H14	119.4
C6—C1—C2	119.7 (12)	C9—C14—H14	119.4
C7—C1—C2	121.9 (11)	N2—C15—C9	127.5 (11)
C15—N2—C20	116.4 (9)	N2—C15—H15	116.3
C15—N2—Mn1	124.1 (7)	C9—C15—H15	116.3
C20—N2—Mn1	119.4 (7)	O4—C16—H16A	109.5
C3—O2—C8	117.0 (11)	O4—C16—H16B	109.5
O1—C2—C3	121.0 (12)	H16A—C16—H16B	109.5
O1—C2—C1	120.9 (10)	O4—C16—H16C	109.5
C3—C2—C1	118.1 (12)	H16A—C16—H16C	109.5
C10—O3—Mn1	130.1 (8)	H16B—C16—H16C	109.5
C4—C3—C2	122.2 (15)	N1—C17—C18	107.4 (9)
C4—C3—O2	123.9 (11)	N1—C17—H17A	110.2
C2—C3—O2	113.8 (11)	C18—C17—H17A	110.2
C16—O4—C11	118.0 (12)	N1—C17—H17B	110.2
C3—C4—C5	118.7 (13)	C18—C17—H17B	110.2
C3—C4—H4	120.7	H17A—C17—H17B	108.5
C5—C4—H4	120.7	O5—C18—C17	110.1 (9)
C18—O5—Mn1^i^	122.9 (7)	O5—C18—H18A	109.6
C18—O5—H5	109.4	C17—C18—H18A	109.6
Mn1^i^—O5—H5	127.6	O5—C18—H18B	109.6
C6—C5—C4	121.1 (12)	C17—C18—H18B	109.6
C6—C5—H5A	119.4	H18A—C18—H18B	108.2
C4—C5—H5A	119.4	O6—C19—C20	112.1 (10)
C19—O6—Mn1^ii^	124.6 (8)	O6—C19—H19A	109.2
C19—O6—H6	105.1	C20—C19—H19A	109.2
Mn1^ii^—O6—H6	122.3	O6—C19—H19B	109.2
C5—C6—C1	119.9 (13)	C20—C19—H19B	109.2
C5—C6—H6A	120.0	H19A—C19—H19B	107.9
C1—C6—H6A	120.0	C19—C20—N2	113.9 (9)
N1—C7—C1	127.1 (11)	C19—C20—H20A	108.8
N1—C7—H7	116.5	N2—C20—H20A	108.8
C1—C7—H7	116.5	C19—C20—H20B	108.8
O2—C8—H8A	109.5	N2—C20—H20B	108.8
O2—C8—H8B	109.5	H20A—C20—H20B	107.7
H8A—C8—H8B	109.5		
			
O3—Mn1—O1—C2	−95 (59)	C8—O2—C3—C2	151.0 (12)
N1—Mn1—O1—C2	−10.8 (10)	C2—C3—C4—C5	−4 (2)
N2—Mn1—O1—C2	170.0 (11)	O2—C3—C4—C5	177.9 (11)
O6^i^—Mn1—O1—C2	−103.2 (10)	C3—C4—C5—C6	4 (2)
O5^ii^—Mn1—O1—C2	77.5 (10)	C4—C5—C6—C1	−4 (2)
O3—Mn1—N1—C7	−176.6 (9)	C7—C1—C6—C5	−172.8 (12)
O1—Mn1—N1—C7	3.9 (9)	C2—C1—C6—C5	4.3 (19)
N2—Mn1—N1—C7	96 (30)	C17—N1—C7—C1	−177.0 (11)
O6^i^—Mn1—N1—C7	93.7 (8)	Mn1—N1—C7—C1	−1.4 (16)
O5^ii^—Mn1—N1—C7	−85.6 (9)	C6—C1—C7—N1	179.4 (12)
O3—Mn1—N1—C17	−1.3 (8)	C2—C1—C7—N1	2.3 (18)
O1—Mn1—N1—C17	179.1 (8)	Mn1—O3—C10—C9	−5.3 (18)
N2—Mn1—N1—C17	−89 (30)	Mn1—O3—C10—C11	168.8 (8)
O6^i^—Mn1—N1—C17	−91.1 (8)	C14—C9—C10—O3	179.7 (12)
O5^ii^—Mn1—N1—C17	89.7 (8)	C15—C9—C10—O3	−1.5 (18)
O3—Mn1—N2—C15	−1.5 (10)	C14—C9—C10—C11	5.7 (17)
O1—Mn1—N2—C15	178.0 (10)	C15—C9—C10—C11	−175.6 (11)
N1—Mn1—N2—C15	86 (30)	C16—O4—C11—C12	28.4 (19)
O6^i^—Mn1—N2—C15	88.2 (10)	C16—O4—C11—C10	−151.5 (12)
O5^ii^—Mn1—N2—C15	−92.5 (10)	O3—C10—C11—C12	177.8 (11)
O3—Mn1—N2—C20	175.9 (8)	C9—C10—C11—C12	−7.4 (18)
O1—Mn1—N2—C20	−4.6 (8)	O3—C10—C11—O4	−2.3 (15)
N1—Mn1—N2—C20	−97 (30)	C9—C10—C11—O4	172.5 (10)
O6^i^—Mn1—N2—C20	−94.4 (8)	O4—C11—C12—C13	−177.0 (12)
O5^ii^—Mn1—N2—C20	84.8 (8)	C10—C11—C12—C13	3 (2)
Mn1—O1—C2—C3	−166.4 (9)	C11—C12—C13—C14	3 (2)
Mn1—O1—C2—C1	14.1 (16)	C12—C13—C14—C9	−5 (2)
C6—C1—C2—O1	175.1 (11)	C10—C9—C14—C13	0 (2)
C7—C1—C2—O1	−7.9 (17)	C15—C9—C14—C13	−178.4 (13)
C6—C1—C2—C3	−4.5 (17)	C20—N2—C15—C9	178.8 (11)
C7—C1—C2—C3	172.5 (12)	Mn1—N2—C15—C9	−3.7 (18)
O1—Mn1—O3—C10	−90 (59)	C10—C9—C15—N2	6.1 (19)
N1—Mn1—O3—C10	−173.4 (10)	C14—C9—C15—N2	−175.1 (13)
N2—Mn1—O3—C10	5.8 (10)	C7—N1—C17—C18	−103.3 (12)
O6^i^—Mn1—O3—C10	−80.9 (10)	Mn1—N1—C17—C18	81.0 (9)
O5^ii^—Mn1—O3—C10	98.4 (10)	Mn1^i^—O5—C18—C17	−162.6 (6)
O1—C2—C3—C4	−175.0 (12)	N1—C17—C18—O5	70.0 (12)
C1—C2—C3—C4	4.6 (19)	Mn1^ii^—O6—C19—C20	159.0 (7)
O1—C2—C3—O2	2.9 (17)	O6—C19—C20—N2	−65.1 (13)
C1—C2—C3—O2	−177.5 (11)	C15—N2—C20—C19	97.8 (13)
C8—O2—C3—C4	−31.1 (19)	Mn1—N2—C20—C19	−79.8 (11)

Symmetry codes: (i) *x*, *y*+1, *z*; (ii) *x*, *y*−1, *z*.

### Hydrogen-bond geometry (Å, º)

**Table:**

*D*—H···*A*	*D*—H	H···*A*	*D*···*A*	*D*—H···*A*
O5—H5···I1*A*^iii^	0.86	2.86	3.488 (8)	131

Symmetry code: (iii) −*x*, −*y*+1, *z*−1/2.
